# RG203KR Mutations in SARS-CoV-2 Nucleocapsid: Assessing the Impact Using a Virus-Like Particle Model System

**DOI:** 10.1128/spectrum.00781-22

**Published:** 2022-07-06

**Authors:** Harsha Raheja, Soma Das, Anindita Banerjee, Dikshaya P., Deepika C., Debanjan Mukhopadhyay, Subbaraya G. Ramachandra, Saumitra Das

**Affiliations:** a Department of Microbiology and Cell Biology, Indian Institute of Science, Bangalore, India; b Department of Biochemistry, Indian Institute of Science, Bangalore, India; c National Institute of Biomedical Genomics, Kalyani, India; d Central Animal Facility, Indian Institute of Science, Bangalore, India; Karolinska Institutet

**Keywords:** SARS-CoV-2, VLP, vaccine

## Abstract

The emergence and evolution of SARS-CoV-2 is characterized by the occurrence of diverse sets of mutations that affect virus characteristics, including transmissibility and antigenicity. Recent studies have focused mostly on spike protein mutations; however, SARS-CoV-2 variants of interest (VoI) or concern (VoC) contain significant mutations in the nucleocapsid protein as well. To study the relevance of mutations at the virion level, recombinant baculovirus expression system-based virus-like particles (VLPs) were generated for the prototype Wuhan sequence along with spike protein mutants like D614G and G1124V and the significant RG203KR mutation in nucleocapsid. All four structural proteins were assembled in a particle for which the morphology and size, confirmed by transmission electron microscopy, closely resembled that of the native virion. The VLP harboring RG203KR mutations in nucleocapsid exhibited augmentation of humoral immune responses and enhanced neutralization by immunized mouse sera. Results demonstrate a noninfectious platform to quickly assess the implication of mutations in structural proteins of the emerging variant.

**IMPORTANCE** Since its origin in late 2019, the SARS-CoV-2 virus has been constantly mutating and evolving. Current studies mostly employ spike protein (S) pseudovirus systems to determine the effects of mutations on the infectivity and immunogenicity of variants. Despite its functional importance and emergence as a mutational hot spot, the nucleocapsid (N) protein has not been widely studied. The generation of SARS-CoV-2 VLPs in a baculoviral system in this study, with mutations in the S and N proteins, allowed examination of the involvement of all the structural proteins involved in viral entry and eliciting an immune response. This approach provides a platform to study the effect of mutations in structural proteins of SARS-CoV-2 that potentially contribute to cell infectivity, immune response, and immune evasion, bypassing the use of infectious virus for the same analyses.

## INTRODUCTION

COVID-19 has been one of the leading causes of death globally since its emergence in December 2019. The coronavirus SARS-CoV-2 has been identified as the causative agent, and its spread has stretched the capacities of health care systems and negatively affected the global economy. SARS-CoV-2 has a 30-kb single-stranded genome encoding 4 structural and 16 nonstructural proteins (1). It is closely related to severe acute respiratory syndrome coronavirus (SARS) and Middle Eastern respiratory syndrome virus (MERS), which have caused epidemics in the recent past. During infection, SARS-CoV-2 virus enters cells through the ACE-2 receptor (present on the epithelial cells lining the respiratory tract), which is recognized specifically by the spike protein of SARS-CoV-2 virus (2). Along with the spike protein (S), the envelope (E) and membrane (M) glycoproteins together form the virion structure which surrounds the genomic RNA coated by the nucleocapsid (N) protein. Studies have shown that these structural proteins elicit host immune responses, resulting in a host generating specific antibodies against them (3, 4). Nucleocapsid has been shown to be highly immunogenic and a promising vaccine target in SARS-CoV infection as well (5, 6).

New virus variants with mutations in these proteins are emerging continuously, with increased transmissibility and severity. It is of utmost importance to understand the molecular basis and effects of these mutations for effective therapeutics and vaccine development. However, generating mutant infectious clones of SARS-CoV-2 and studying their effects on immunogenicity, pathogenicity, and transmissibility are technically challenging because of the requirement of biosafety level 3 (BSL-3) protections for working with the virus. We have designed a virus-like particle (VLP), which is composed of all the structural proteins that form noninfectious virus-like particles but generates immune responses similar to those for infectious virus particles, enabling the study of mutations of all the structural proteins in a more physiologically relevant system. VLPs are produced using baculovirus-mediated gene expression, because of this system’s advantages over adenovirus and lentivirus systems (7, 8). The mutations D614G and G1124V within spike protein and RG203KR within the nucleocapsid revealed plausible structural implications, as depicted in previous studies (9, 10). D614G predominantly circulated worldwide and is presently incorporated into the backbone of all emerging strains (variants of concern [VoCs] and variants of interest [VoIs]). Structural analysis has shown that this mutation alters the conformation of spike protein, promoting the receptor binding domain (RBD) in an “up” state and providing enhanced furin cleavage efficiency (11). Clinical evidence has revealed an increase in viral replication in the upper respiratory tract by augmenting infectivity and virion stability (12). G1124V is one of the major mutations on CD8 T-cell epitopes of the S protein that might have significant implications in a context of immunogenicity.

The R203K and G204R mutations in nucleocapsid were first identified in the A2a lineage within China and subsequently have spread within other lineages across Western Europe, the United Kingdom, and then to the United States and other parts of the world through a number of VoCs and VoIs, *viz.*, the Alpha, Gamma, Lambda variants and now, in the most underscored VoC, the Omicron variant. This RG203KR mutation has been shown to enhance infectivity, fitness, and virulence (13–15). Recently, a different VLP approach involving incorporation of coding RNA in VLPs has been used to study the effect of nucleocapsid mutations on transmissibility of the virus (16). However, the impact on immunogenicity remains to be studied. Here, we have incorporated these mutations to study their impact, using VLP as a platform.

## RESULTS

### Expression of SARS-CoV-2 VLPs in Sf21 cells.

**FIG 1:**
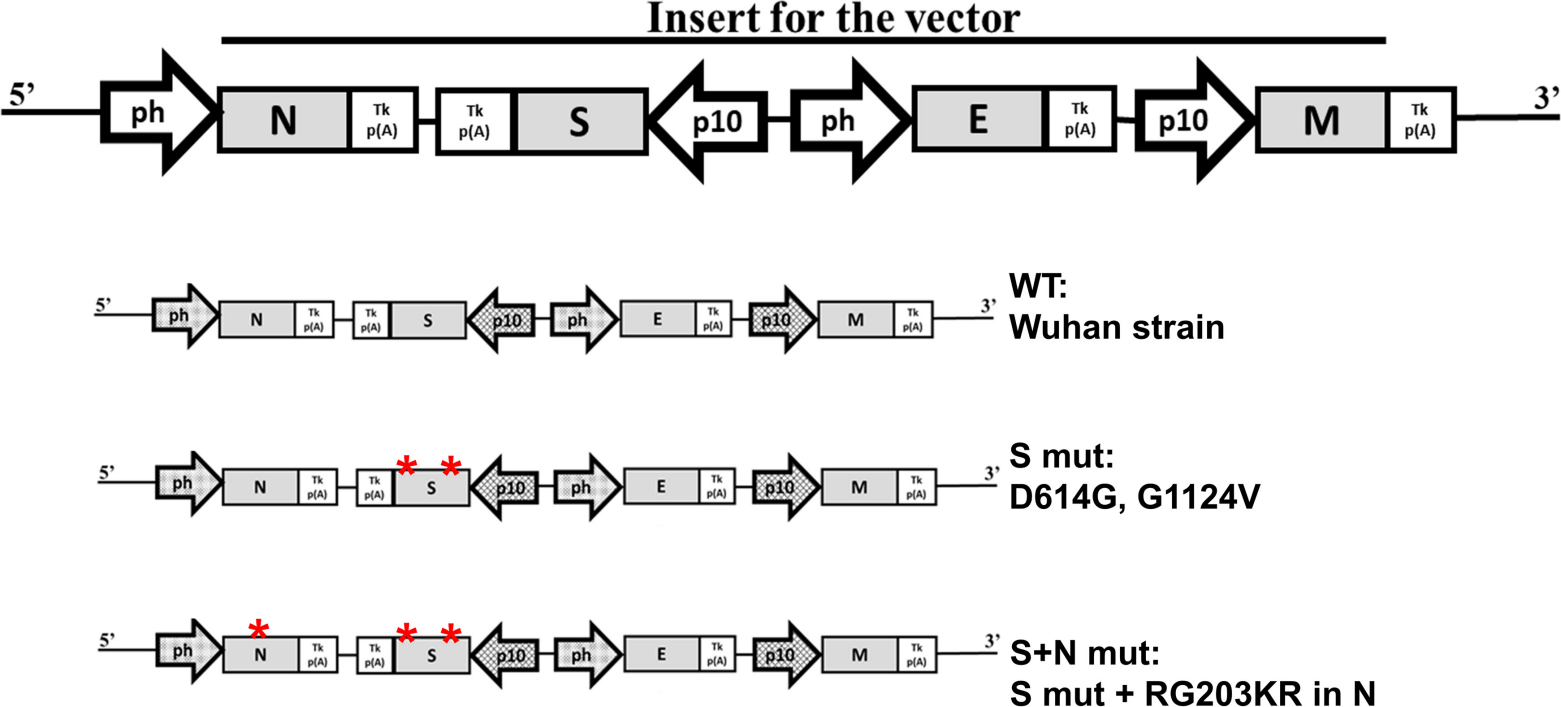
Schematic for SARS-CoV-2 VLP expression construct. ph, baculovirus polyhedrin promoter; p10, baculoviral p10 promoter; S, SARS-CoV-2 spike protein; E, SARS-CoV-2 envelope protein; M, SARS-CoV-2 membrane protein; N, SARS-CoV-2 nucleocapsid protein; Tk p(A), Thymidine kinase Poly-adenylation sequence.

Viral structural proteins form the viral capsid and envelope. SARS-CoV-2 codes for 4 structural proteins, namely, nucleocapsid (N) protein, which coats the viral genetic RNA, spike (S) protein, which creates the viral capsid, membrane (M) protein, which interacts with all the other structural proteins and defines the shape of the viral envelope, and envelope (E) protein, which makes the viral envelope. We have expressed all four structural proteins of SARS-CoV-2 in a baculovirus expression system to form VLPs. Generally, the viral proteins are produced as polyproteins which are later cleaved by viral and host proteases. In contrast, SARS-CoV-2 forms separate mRNAs for expressing each structural protein. To achieve similar expression, we synthesized (Genscript, USA) all 4 structural proteins with different promoters in the baculovirus expression vector (TaKaRa, USA). The schematic for the order of gene arrangement is shown in Fig. 1. The plasmid was cotransfected along with Bsu36I-digested baculovirus DNA, BacPAK6. Recombination sites are present in the BacPAK9 plasmid around the site of insertion. Recombination of SARS-CoV2 genes encoding plasmid with the viral DNA resulted in the formation of a baculovirus expressing all four proteins to form the VLP. Viral RNA keeps accumulating mutations as it propagates. Earlier, we reported the emergence of mutations in S- and N-coding genes (9). Based on this, we generated three VLP constructs. The first one contains sequences of the original Wuhan strain as prototype (WT-VLP). The second one harbors D614G and G1124V mutations in the S protein (S mut-VLP). The third one harbors an RG203KR mutation in the N protein along with the previous S mutations (S+N mut-VLP).

### Purification and characterization of VLPs.

**FIG 2:**
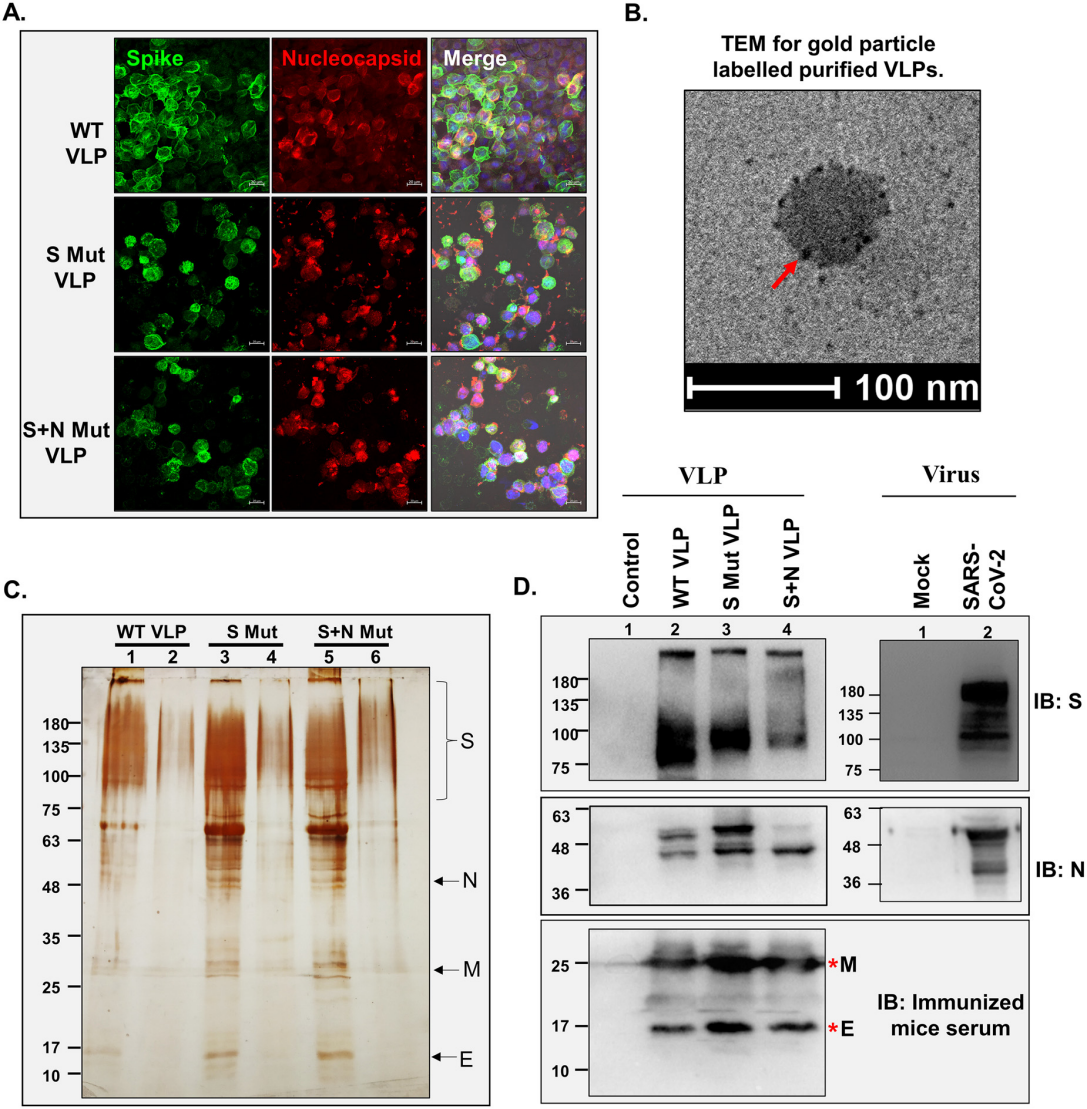
Characterization of SARS-CoV-2 VLPs. (A) Expression of SARS-CoV-2 proteins in Sf21 cells. Baculovirus-infected Sf21 cells were harvested after 96 h and processed for confocal staining using anti-S- and anti-N-specific primary antibodies and Alexa Fluor 488 (AF488)- and AF633-labeled secondary antibodies. Nuclei were counterstained using DAPI. Bar, 20 μm. (B) Transmission electron microscope images of purified VLPs. The purified VLPs were fixed, added to the copper grid, and stained for S protein using specific primary and immunogold-labeled secondary antibody. Negative staining was done using uranyl oxalate. The arrow indicates immunogold-labeled S protein. (C) The purified VLPs were loaded onto SDS–10% polyacrylamide gels, followed by silver staining (adjacent to the VLP lane, a lower fraction of the gradient was loaded, confirming the VLP purity). Lanes 1, 3, and 5 represent purified VLP lanes. (D) Western blot to detect the presence of S protein and N protein using specific primary antibodies and HRP-tagged secondary antibodies for purified VLPs and SARS-CoV-2-infected cell lysate. Sera from VLP-injected mice was used as primary antibody, followed by HRP-tagged anti-mouse antibody as secondary antibody for detecting M protein and E protein.

After baculovirus titration, the expression of SARS-CoV-2 proteins through recombinant baculovirus was confirmed 3 to 4 days postransduction of Sf21 cells by immunofluorescence (Fig. 2A). Coexpression of both the proteins in the same cells confirmed the production of VLP proteins through recombinant baculovirus. VLPs were purified by overlaying the cell lysate 96 h postransduction on a 20 to 50% (wt/wt) sucrose gradient, followed by ultracentrifugation at 28,000 rpm for 3 h. The VLP-containing band was used for characterization. Transmission electron microscopy (TEM), involving negative staining and immunogold labeling for S protein, revealed the particle diameter ranged from 30 to 100 nm (Fig. 2B). VLP purity was assessed by silver staining of the samples run on SDS-PAGE gels (Fig. 2C). Four prominent bands around the expected size of S (150 to 180 kDa), E (12 kDa), M (26 kDa), and N (48 to 49 kDa) proteins were observed, and these data were further confirmed by Western blotting with the corresponding antibodies (Fig. 2D). To compare VLPs with the actual virus protein, virus-infected cell lysate was loaded and probed with the same antibodies. Bands at the same size for both VLP and virus-infected lysate confirmed similar expression levels of proteins in the two systems. Since anti-M and anti-E antibodies were not available commercially, sera obtained from mice injected with VLPs was used to probe the blots. These assays show the purity of the VLP isolated from the baculovirus expression platform.

### VLP entry into cells.

**FIG 3:**
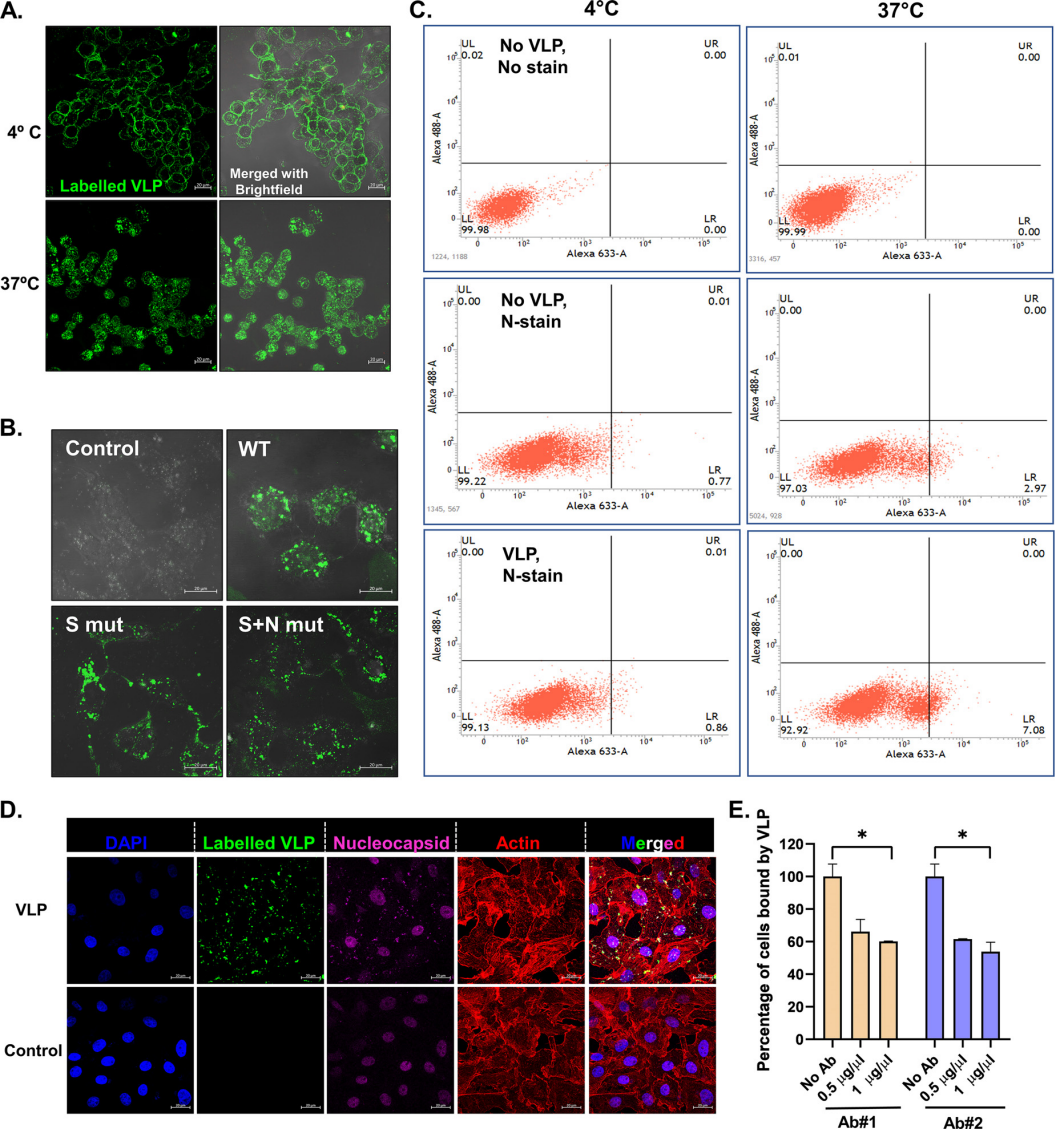
VLP binding and entry into cells. (A) Dynamics of VLP binding to cells. AF488-labeled VLPs were incubated with Vero cells at the indicated temperature, and VLP binding was visualized by confocal imaging. Bar, 20 μm. (B) Vero cells were incubated with AF488-labeled VLPs for 2 h and processed for confocal imaging. Bar, 20 μm. (C) Unlabeled VLPs were incubated with Vero cells at the indicated temperatures for 2 h, followed by immunostaining using nucleocapsid (N) protein-specific primary antibody and AF633-labeled secondary antibody. The percentage of cells bound by N antibody was quantified by flow cytometry. (D) AF488-labeled VLPs were incubated with Vero cells at 37°C for 2 h, followed by immunostaining using nucleocapsid (N) protein-specific primary antibody and AF633-labeled secondary antibody. Nucleus was counterstained with DAPI, and the cell boundary was visualized with rhodamine-phalloidin. Bar, 20 μm. (E) Labeled VLPs were incubated with the indicated concentration of commercial antibodies against S protein prior to binding with Vero cells. VLP binding to cells after preincubation was analyzed by flow cytometry and quantified. Blue and pink colors represent the two different antibodies used. Student’s *t* test was used for statistical analysis. Error bars represent standard errors of the means. *, *P* < 0.05; **, *P* < 0.01; ***, *P* < 0.001.

To assess the physiological binding of VLPs to the ACE-2 receptor, Vero E6 cells were used. The VLPs were fluorescently labeled *in vitro* with Alexa Fluor 488, and the binding of labeled VLPs to Vero E6 cells was assayed. U937 cells, which lack an ACE-2 receptor, were used as negative controls in the assay. Dose-dependent binding to Vero cells and absence of VLP binding to U937 cells confirmed the specificity of VLP binding to ACE-2-expressing cells (data not shown). To study the dynamics of cell entry, VLPs were incubated with the Vero cells at either 4°C or 37°C for 2 h. Results showed that VLPs bound to the cell surface at 4°C and internalized at 37°C (Fig. 3A). The binding and internalization of all 3 VLPs were visualized by confocal microscopy at higher magnification (Fig. 3B). To investigate whether VLP binding was similar to the actual virus binding and internalization, flow cytometry was performed. It appeared that when the VLPs were bound on the surface of cells, the N protein was not accessible to the antibody against it, and thus we were not able to detect the staining of nucleocapsid when VLPs were incubated at 4°C (0.86%). However, this was altered at 37°C, wherein the VLP entered the cells and N protein was released from the particle, becoming accessible to the antibodies against it (7.08%) (Fig. 3C). The accessibility of N protein after VLP internalization was confirmed by immunofluorescence imaging (Fig. 3D). This depicts a mechanism similar to the virus entry. This was further confirmed by inhibition of VLP attachment and entry to the cells in the presence of antibodies against spike protein (Fig. 3E), which would block the viral protein for interaction with the cell surface receptor. The results provide proof of concept on the potential use of VLPs as a tool to study the entry mechanism of virus.

### VLP-induced toxicity analysis in mice.

**FIG 4:**
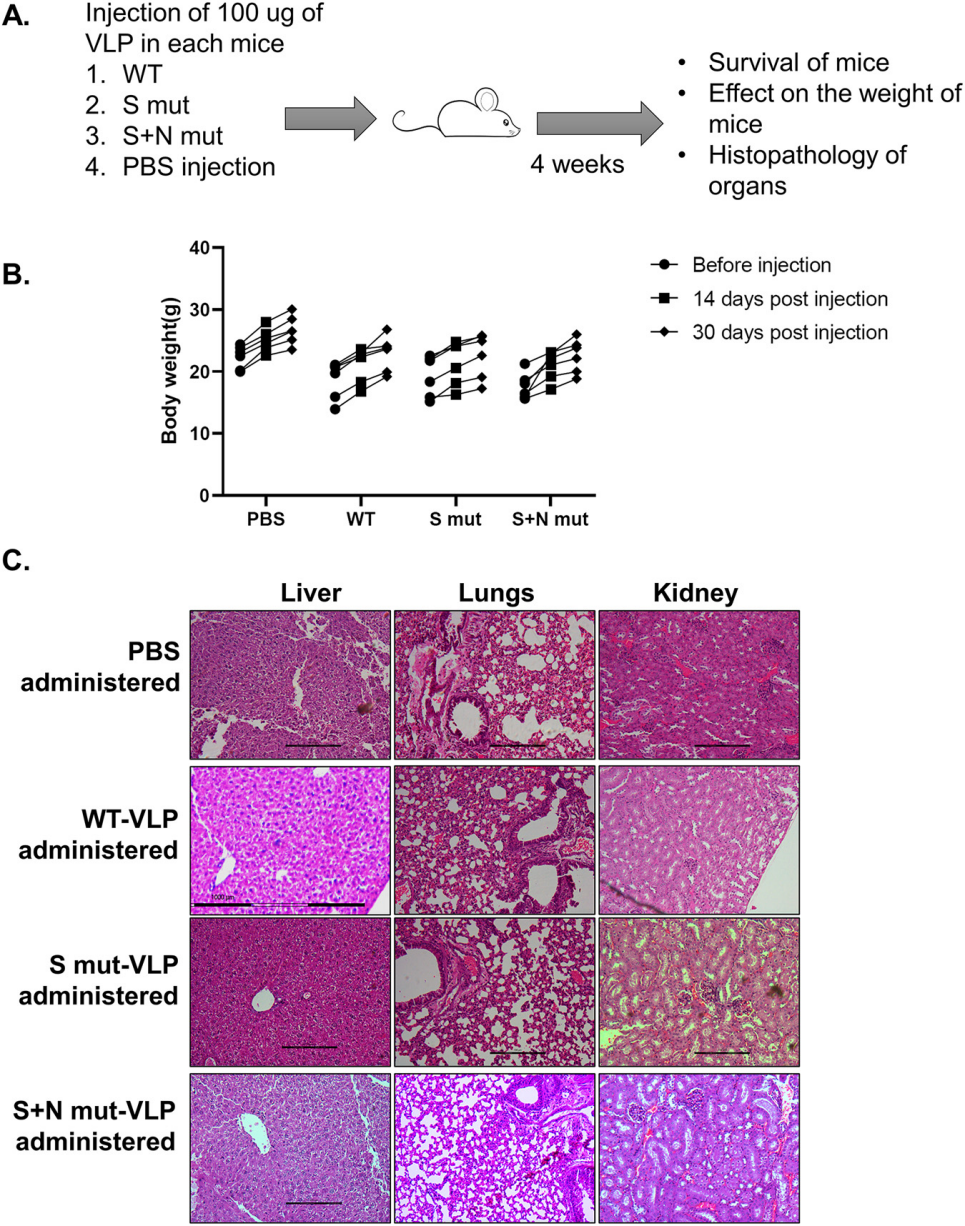
VLP toxicity analysis in mice. (A) Workflow for VLP toxicity analysis in mice. (B) Body weight of mice was recorded at the mentioned time points after VLP administration. Six animals were included for each group. Each dot represents a different animal. (C) Light microscopy images of liver, lungs, and kidney sections of PBS-treated or respective VLP-treated mice after hematoxylin and eosin staining of the sections. The images are representative of one animal from each group.

To assess if the VLP can be used as an immunogen, we first checked its tolerance in mice by injecting a high dose (100 μg) into 6-week-old BALB/c mice. We took 4 groups of mice, one for each VLP and one for the vehicle control (phosphate-buffered saline [PBS]), with 6 mice in each group. The mice were monitored for 4 weeks for appearance of any signs of toxicity (Fig. 4A). All the mice survived with no effect on increase in body weight (Fig. 4B). Additionally, administration of purified VLPs did not affect the histology of mouse liver, kidney, or lungs, as observed by histopathology (Fig. 4C).

### VLP-induced immune response in mice.

**FIG 5:**
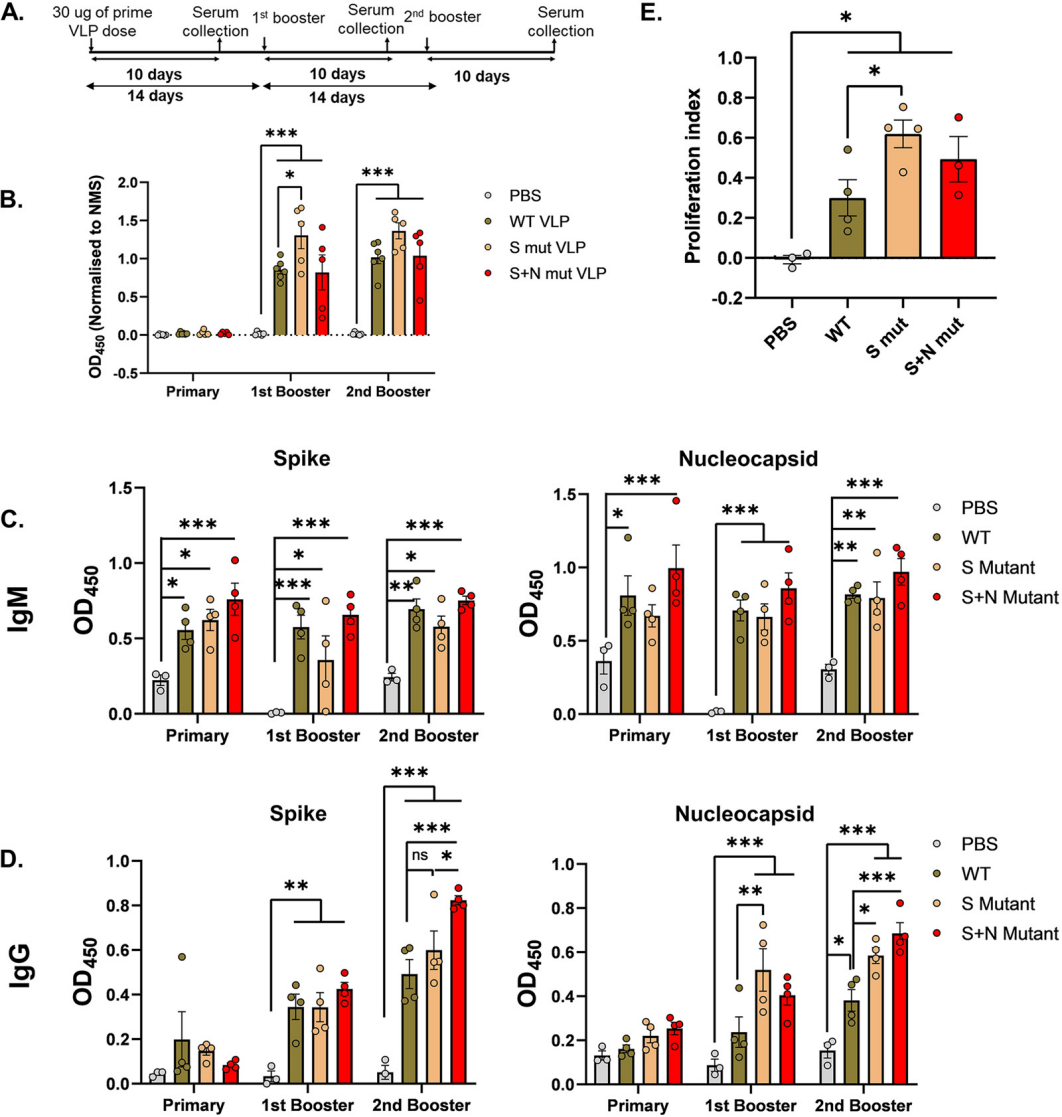
Immune response against SARS-CoV-2 VLP injection in mice. (A) Schematic of immunogenicity studies in mice. ELISA was performed with murine sera collected after immunization with the indicated VLPs at different time points. (B to D) WT-VLP or full-length spike protein or nucleocapsid protein were used as antigens. Mouse serum samples were added to the coated antigens and either HRP-tagged IgG and IgM (B) or biotin-labeled IgM and IgG antibodies (C and D) were used as secondary antibodies. Color development by streptavidin-HRP followed by addition of TMB substrate was quantified and plotted after normalization as described previously. Sera from 3 animals for PBS injection and 4 animals each for the VLP injection were analyzed by ELISA. Each point represents the value obtained from individual mouse serum. Two-way ANOVA was done for statistical analysis. Error bars represent SEM. *, *P* < 0.05; **, *P* < 0.01; ***, *P* < 0.001. (E) Splenocyte proliferation in response to peptides against spike protein was quantified in an MTT assay. MTT was added after 24 h of peptide stimulation, and color development was quantified and plotted. Each point represents splenocyte proliferation from a different mouse. Student’s *t* test was used for statistical analysis. Error bars represent SEM. *, *P* < 0.05; **, *P* < 0.01; ***, *P* < 0.001.

To assess their immunogenicity, the purified VLPs were injected into mice and sera were collected as per the timeline mentioned in Fig. 5A. We took 4 groups of mice, one for each VLP and one for the vehicle control (PBS), with 6 mice in each group. Humoral responses generated against the WT-VLP were quantified in an enzyme-linked immunosorbent assay (ELISA), and all three sera from VLP-injected mice exhibited a significant response compared to vehicle control. Grossly, immunization with S mut-VLP elicited a higher response after the booster dose than WT-VLP or even S+N mut-VLP (Fig. 5B). Humoral immunity in the form of IgM (first class of antibodies) and IgG (antibody produced after class switching) against total S protein and nucleocapsid was measured (Fig. 5C and D). Immunization with VLPs elicited a strong IgM and IgG response against spike and nucleocapsid proteins. The highest levels of IgM against both the antigens were observed when animals were immunized with the S+N mut-VLP. The IgG response against spike and nucleocapsid increased from first booster to second booster. The effect of mutations was pronounced after the second booster, where we observed that the S+N-mut sera demonstrated a significantly higher IgG response for spike than did the WT-VLP sera. The effect of mutations on the immunogenicity against N was evident in the sera obtained after the first booster as well as second booster. After the first booster, S mut-VLP immunization showed the highest response against N, with no significant differences between responses to WT-VLP and S+N mut-VLP in the corresponding immunized mouse sera. For the IgG response against nucleocapsid after the second booster, again there was a heightened response in sera from S mut-VLP injected animals, and the incorporation of additional N mutation further increased the response. It appears that the RG203KR mutation in nucleocapsid gradually increases the IgG response against S and N proteins, which indicates that the mutation in nucleocapsid can potentially alter the viral structure, especially the antigenic sites. To further assess the T-cell response against the injected VLPs, proliferation of T cells in response to *in vitro* stimulation with peptides against the S protein was measured in an 3-(4,5-dimethylthiazol-2-yl)-2,5-diphenyltetrazolium bromide (MTT) assay. A significant difference in proliferation of T cells isolated from VLP-injected mouse spleen compared to vehicle control established the specific activation and proliferation of T cells by the injected VLP (Fig. 5E). Among the VLPs, as with the humoral response, mutant VLPs showed higher T-cell proliferation than did WT-VLP. These assays depict the utilization of VLP to study the altered immunogenicity upon the incorporation of spike and nucleocapsid mutations.

### Applications of the VLPs and associated mutations.

**FIG 6:**
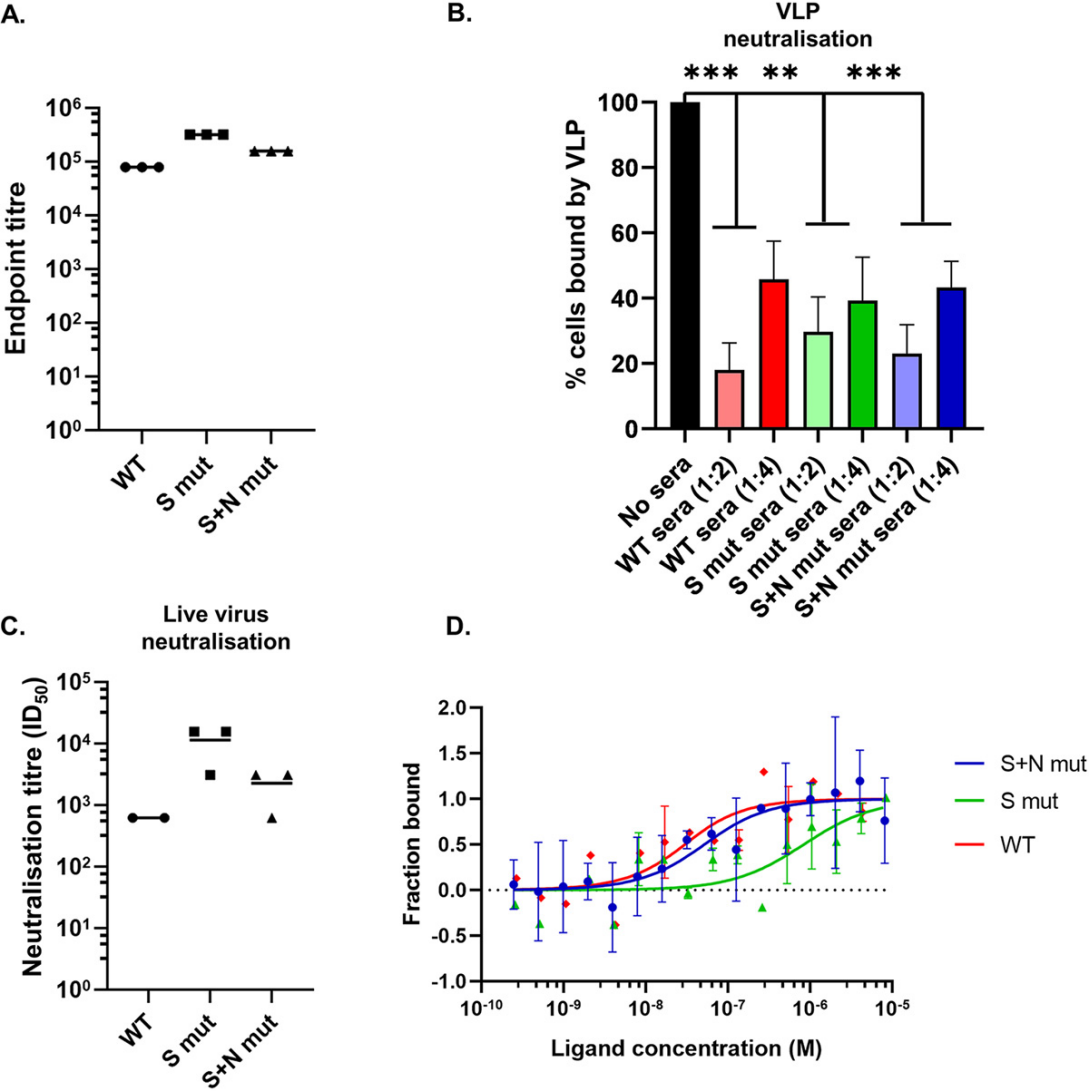
Applications of the VLP system with incorporated mutations. (A) Endpoint titration of sera from VLP-immunized animals. ELISA plates were coated with WT-VLP, and one representative serum sample from each VLP-immunized mouse was used to calculate the endpoint titer. (B) Neutralization of VLP binding to cells. Labeled VLPs were incubated with the indicated dilutions of sera prior to binding with Vero cells. VLP binding to cells after preincubation was analyzed by flow cytometry and quantified. Student’s *t* test was used for statistical analysis. Error bars represent SEM. *, *P* < 0.05; **, *P* < 0.01; ***, *P* < 0.001. (C) Live virus neutralization using sera from VLP-immunized animals. Virus was incubated with heat-inactivated sera from VLP-immunized animals prior to attachment to Vero cells. The serum dilution which resulted in a 50% reduction of plaque numbers compared to preincubation with serum from PBS-immunized animals was plotted. Each point represents a different mouse serum. (D) Binding affinity of VLPs with recombinant ACE-2 was calculated using microscale thermophoresis.

The endpoint titer of immunized sera was calculated using one serum sample from each group. We observed a high endpoint titer of 10^5^ to 10^6^ against all 3 VLPs (Fig. 6A). This is comparable and even higher than the titers obtained upon pseudovirus immunization in mice (17) and hence points toward the potential usage of VLP as an efficient immunogenic system. The highest-titer sera obtained upon immunization with VLPs were further used to check the neutralization of labeled VLP binding to Vero cells. We observed more than 50% VLP neutralization in the presence of a 1:2 dilution of sera from mice immunized with all 3 VLPs (Fig. 6B). The immunized sera were further checked for the ability to neutralize infectious SARS-CoV-2 virus. Neutralization 50% infectious doses (ID_50_s) of 10^3^ to 10^4^ signified the efficient production of neutralizing antibodies upon VLP immunization (Fig. 6C).

The binding affinity of WT and mutant VLPs was calculated using microscale thermophoresis (MST) (Fig. 6D). We found that the WT-VLP bound to recombinant ACE-2 with a *K_d_* of 19.9 ± 7.8 nM. Incorporation of D614G in combination with G1124V (S mut-VLP) led to decreased affinity, with a *K_d_* of 827.9 ± 82.3 nM. Interestingly, addition of the RG203KR mutation in this background (S+N mut-VLP) restored the binding affinity to a *K_d_* of 41.8 ± 3.5 nM. This finding supports the previous report suggesting that the RG203KR mutation in N increases the binding of virus to cells (13). The results justify the use of VLP as a powerful tool to study the impact of emerging viral mutations.

We have illustrated the establishment of a VLP system and its potential use to study the impact of mutations in structural proteins.

## DISCUSSION

SARS-CoV-2 has been one of the leading causes of death worldwide for the past 2 years. New variants are emerging with altered immunogenicity and pathogenicity. One of the major hurdles in detailed studies of this virus is the requirement of a BSL-3 facility. Here, we provide a virus-like particle-based platform which bypasses the requirement to work with infectious virus to study the implications of mutations in the viral structural proteins. We have designed (Fig. 1) and purified baculovirus-based VLPs (Fig. 2) which are noninfectious, safe to handle, and easy to produce in large quantities. The VLP model showed entry kinetics similar to that of the actual virus (Fig. 3). It did not show toxicity in a mouse model (Fig. 4) and proved to be an excellent immunogen to trigger humoral and T-cell responses (Fig. 5). Serum from VLP-immunized animals was able to neutralize live virus and also served as a tool to study the impact of mutations on viral affinity for the ACE-2 receptor (Fig. 6). The differential immune response generated in response to either WT or mutant VLP administration has been assessed using this model.

The incorporated mutations in the VLP (D614G and G1124V within spike, RG203KR within nucleocapsid) revealed the structural significance of these proteins, as proposed in previous studies (9). The RG203KR mutation contributed to alteration in the conformational entropy of the N protein, whereas the D614G mutation in spike could significantly compromise the structural integrity of the prefusion state of the spike protein through increased local conformational entropy. The amino acid G at position 1124 is embedded in the connector domain of the S protein is crucial for spike trimerization and aids in stabilizing the protein conformation during fusion with the host cell membrane. G1124V is one of the major mutations on CD8 T-cell epitopes in S protein which might have significant implications in the context of immunogenicity. The G1124V mutation was found to be beneficial for SARS-CoV-2 to evade immune recognition, as it completely compromises the epitope binding affinity of some major HLA alleles. However, the G1124V variant did not become as dominant as D614G. Therefore, it is important to dissect the cause behind this, such as stability of the viral structure and host immunity that might have prevented this variant from standing out further. The G at position 1124 is in close proximity to the region where spike attaches to the viral membrane, and it is highly conserved among the closely related coronaviruses. G imparts higher conformational freedom, which is beneficial for the viral attachment, compared to V in the mutated spike. This might be the reason for the decreased binding affinity of S mut-VLP with ACE-2. The lower binding affinity with ACE-2 and higher induced antibody response could be the reasons for elimination of the G1124V mutation from the VoCs.

Studies have revealed that copy-choice transposition of the transcriptional regulatory sequence ACGAAC, to the end of the serine-arginine (SR)-rich region of nucleocapsid, creates a novel canonical mRNA and gene-specific expression of the N2 dimerization domain. This transposition has been recognized as the 203 to 204 RG-to-KR mutation. During the pandemic, this is the first time a virus has been shown to have created a new canonical gene and mRNA through this mutation, which could modify the biogenesis of mature virion. The change from RG to KR was not seen to impart any benefit to the replication fitness of the virus, but rather it conferred more basicity to the nucleocapsid phosphoprotein. The RG203KR mutation has been shown to increase the infectivity and virulence of the virus. We saw a similar effect of this mutation in the VLP background. Addition of this mutation to S mut-VLP restored the binding affinity to ACE-2.

SARS-CoV-2 spike protein mutation D614G predominantly circulated worldwide and is presently incorporated into the backbone of all emerging strains. It is linked to increased viral replication in the upper respiratory tract. However, detailed functional implications and the impact of this mutation on the efficacy of the leading vaccines remain to be deciphered.

We have shown that VLP administration leads to high-titer and neutralizing antibody production. Currently, pseudoviruses expressing spike protein alone are widely being used for such assays. Nucleocapsid protein has been shown to be highly immunogenic but is being ignored in immunogen preparations. The VLP platform provides an edge over the pseudoviruses by including all 4 structural proteins that form a structure much more closely resembling the actual virus. This also enables study of the mechanism of viral entry of the original and mutant viruses in a more easily used and noninfectious system which can be taken forward to design inhibitory drugs.

While a VLP resembles the actual virus, it lacks the genomic RNA, and hence one could miss out on the effect of mutations on virus structure in the presence of genomic RNA. Comparison of the morphology and structure of the VLP with the real SARS-CoV-2 virus was not possible due to the inaccessibility of required equipment in a BSL-3 facility.

Because it is nonreplicating, the VLP cannot be used to study virus pathogenesis. Also, the impact of mutations in nonstructural proteins cannot be studied in a VLP system, but it still provides an excellent system to study emerging mutations and the biology of the virus before getting access to infectious virus. The protein sequences of structural proteins can easily be tinkered with to study their impact without the risk of development of highly infectious strains or accidental leakage.

Taken together, we provide a comprehensive report of the impact of the RG203KR mutation in nucleocapsid on the immunogenicity, receptor-binding affinity, and neutralization efficiency by using a model which can be easily manipulated and exploited to study the emerging SARS-CoV-2 mutations in a system closely resembling the virus while being noninfectious. It can be used to study the immune evasion capability of emerging viral variants and the efficacy of administered vaccines against those mutants. The mutations in structural proteins can be easily incorporated in the VLP system and can be used to check the neutralization efficacy of sera from vaccinated individuals.

## MATERIALS AND METHODS

### Cloning, transfection, and generation of SARS-CoV-2 VLPs.

The structural genes S, E, M, and N with respective promoters were synthesized commercially (GenScript, USA) and cloned in pBacPAK9 with restriction sites BamHI and EcoRI. The sequence for gene synthesis were taken from GISAID ID: EPI_ISL_424357 (isolate from Beijing/Wuhan). Vector DNA pBacPAK9 containing the target gene was transfected into Spodoptera frugiperda cells, along with Bsu36I-digested BacPAK6 viral DNA as described earlier (TaKaRa Bio Inc., USA). Briefly, 1 × 10^6^ cells in 35-mm tissue culture dishes was incubated at 27°C for 1 to 2 h, washed with plain medium, and transfected with a mixture containing DNA (100 ng/μL), Bsu36I-digested BacPAK6 viral DNA, and Bacfectin and kept at 27°C for 5 h. A 2% TC100 (Sigma, USA) medium was added and kept at 27°C. After ~5 days of incubation, the medium, which contained viruses produced by the transfected cells, was collected and stored at 4°C. The titers of the generated baculovirus were determined using a BacPAK baculovirus rapid titer kit (Clontech, USA).

### Immunofluorescence staining.

For immunofluorescence staining of Sf21 cells infected with baculovirus expressing SARS-CoV-2 VLP, cells were seeded on coverslips in a 24-well plate for 14 h, followed by infection with respective baculovirus. After the desired time of infection, cells were washed twice with 1× PBS and fixed using 4% paraformaldehyde at room temperature for 20 min. After permeabilization by 0.1% Triton X-100 for 2 min at room temperature, cells were incubated with 3% bovine serum albumin (BSA) at 37°C for 1 h, followed by incubation with the indicated antibody for 2 h at 4°C and then detection with Alexa 633-conjugated anti-mouse or Alexa 488-conjugated anti-rabbit secondary antibody for 30 min (Invitrogen). Images were taken using a Zeiss microscope, and image analysis was done using the Zeiss LSM or ZEN software tools.

For immunofluorescence staining of VLPs in Vero cells, 1 μg of Alexa Flour 488-labeled VLP was added to Vero cells seeded on coverslips in a 24-well plate. After incubation at the desired temperature for the indicated time, staining for nucleocapsid protein was done using the same protocol as described above for Sf21 cells. Nucleus was stained with 4′,6-diamidino-2-phenylindole (DAPI), and rhodamine-phalloidin was used to stain actin filaments to visualize cell boundaries.

### Transmission electron microscopy (immunogold labeling and negative staining).

The purified VLP was diluted in PBS, fixed with 4% paraformaldehyde, and spotted onto 400-mesh carbon-coated copper grids for 10 min. It was then blocked using 1% BSA for 10 min, followed by incubation with primary antibody against SARS-CoV-2 S protein (catalog number 40592-R001, Sino Biological) for 30 min. Thereafter, PBS wash was done 3 to 5 times and the grid was incubated with gold-conjugated anti-rabbit secondary antibody for 15 min. After 7 to 8 PBS washes, 1% glutaraldehyde was added onto the grid for 5 min to stabilize the immunostaining. Again, PBS wash was done 5 times, and the samples were negatively stained using 2% uranyl oxalate. After thorough PBS washes, the grids were air dried and examined under a transmission electron microscope at 80 kV to visualize the immunogold-labeled VLPs.

### Isolation of VLPs.

The baculovirus-infected Sf21 cells were lysed with TEN buffer (10 mM Tris [pH 7.5], 1.0 mM EDTA, 1.0 M NaCl, 0.1% Triton X-100, 1 mM phenylmethylsulfonyl fluoride). For efficient lysis, the lysates underwent 2 freeze-thaw cycles in liquid nitrogen followed by sonication of 3 s on, 3 s off for 2-min cycles at 40% efficiency setting. The lysates were centrifuged at 3,500 rpm for 30 min at 4°C. After centrifugation, the supernatant was collected and added to the top of a 20%–50% sucrose gradient and centrifuged at 28,000 rpm for 3 h at 4°C in an ultracentrifuge using an SW40 rotor. After centrifugation, the opaque band containing VLPs was collected and processed for characterization.

### Labeling of VLPs.

SARS-CoV-2 VLPs were labeled with Alexa Fluor 488 carboxylic acid, succinimidyl ester (catalog number A200000, Sigma) using size-exclusion chromatography columns. The labeled VLPs were used for binding with Vero cells in flow cytometry and imaging assays. For immunofluorescence imaging, Vero cells were seeded on coverslips in a 24-well plate. Labeled VLPs were added to the cells in Dulbecco’s modified Eagle’s medium (DMEM) and incubated for 1 to 2 h at 37°C. Thereafter, coverslips were mounted on slides and images were taken on a Zeiss710 confocal microscope and analyzed by Zen software tools.

### Western blotting.

Protein concentrations of the extracts were assayed with Bradford reagent (Bio-Rad), and equal amounts of cell extracts were separated by SDS–12% PAGE and transferred onto a nitrocellulose membrane (Sigma). Samples were then analyzed by Western blotting using the desired antibodies anti-SARS-CoV-2 S protein (catalog number 40591-T62, Sino Biologicals), anti-SARS-CoV-2 N protein (catalog number 40143-MM05, Sino-Biologicals), and immunized mouse sera followed by the respective secondary antibodies (horseradish peroxidase-conjugated anti-mouse or anti-rabbit IgG; Sigma). Antibody complexes were detected using Immobilon Western systems (Millipore).

### Animal immunization.

Approval for animal experiments was obtained and the Institutional Animal Ethics Committee Guidelines of India National Law on Animal Care and Use were followed for animal experiments. Twenty-four female BALB/c mice, 6 weeks old, were grouped into four groups, and immunization was given intraperitoneally (i.p.). SARS-CoV-2-like particles (LPs) were conjugated with 2% alhydrogel as an adjuvant for immunization. In the first regimen, 30 μg of SARS-CoV-2-LPs was administered per mouse followed by two boosters with 15 μg SARS-CoV-2-LPs per mouse at an interval of 2 weeks between injection. In addition, a group immunized with PBS served as a negative control. Preimmune sera, before the start of the experiment, and postimmune sera at each booster dose were isolated and stored at −70°C. Mice were sacrificed and spleens were removed on the 10th day after the final booster dose. Splenocytes were isolated as a mixed cell suspension using a 70-μm cell strainer. ACK lysis buffer (155 mM NH_4_Cl, 10 mM KHCO_3_, 0.1 mM EDTA) was used to deplete red blood cells from the cell suspension.

### Toxicity study in mice.

Twenty-four male BALB/c mice (6 weeks old) were grouped into four groups and 100 μg of SARS-CoV-2-LPs conjugated with 2% alhydrogel was administered i.p. The weight and behavior of the animals were monitored for 28 days. After 28 days, the animals were sacrificed and liver, lungs, heart, and kidneys were extracted. To examine the toxicity effect 4 weeks post-SARS-CoV-2-LPs administration in both control and injected groups, the histological analysis of 10% neutral buffered formalin-fixed mouse tissues was performed commercially.

### Measurement of humoral immune response after VLP immunization.

ELISA was performed with murine sera collected after immunization with the indicated VLPs at different time points. SARS-CoV-2 spike protein (S) (catalog number 40589-V08B1, Sino Biological Inc.), nucleocapsid protein (N) (catalog number 40588-V08B, Sino Biological Inc.), and WT-VLP were used for coating the plates. ELISA was performed using Nunc MaxiSorp plates (Thermo Fisher Scientific, USA). Biotinylated goat anti-mouse IgM, IgG, streptavidin-conjugated horseradish peroxidase (Str-HRP), and BSA were purchased from Sigma-Aldrich, USA. 3,3′,5,5′-Tetramethylbenzidine (TMB) solution was purchased from Applied Biological Materials (ABM), Canada. All the rest of the chemicals were purchased from Sisco Research Laboratories (SRL), India, and are of molecular biology grade.

ELISA was performed as described elsewhere (18). Briefly, ELISA plates were coated with 100 ng of recombinant protein or 1 μg of WT-VLP dissolved in PBS overnight. The next day, plates were washed with PBS with 0.05% Tween 20 (wash buffer) three times and subsequently blocked with PBS with 2% BSA and 0.05% Tween 20 (blocking buffer) for 2 h. Thereafter 100 μL murine sera was added in a 1:10,000 dilution in blocking buffer for 1 h for IgG and overnight for IgM. Aliquots of 100 μL of a 1:1,000 dilution of murine sera were used in the ELISA against the WT-VLP. Following that, plates were washed thrice with wash buffer and 100 μL biotinylated goat anti-mouse IgM (1:10,000) or IgG (1:25,000) was added to the wells for 2 more hours. After 2 h, plates were again washed thrice with wash buffer and 100 μL streptavidin-HRP was added for 30 min. Finally, plates were washed five times with wash buffer and 100 μL TMB substrate was added. Following 10 min, 50 μL of stop solution (2 N HCl) was added, and absorbance was recorded using a microplate reader (Spectramax M2e; Molecular Devices, USA) at 450 nm. Statistical analyses were performed using Graph Pad Prism version 8.0. A two-way analysis of variance (ANOVA) followed by Tukey’s multiple comparison test was performed for determination of significance between groups and time points.

To determine the endpoint titers,1:2 increasing dilutions to immunized mouse sera were added to the WT-VLP-coated ELISA plate. After following the whole protocol described above, the highest serum dilution that gave a signal above the cutoff value (the optical density at 405 nm [OD_405_] of negative controls, ±2 standard deviations) was plotted as the endpoint titer.

### Splenocyte proliferation assay.

In a 96-well plate, 10^5^ splenocytes were seeded and stimulated with peptides against S protein (GenScript; 2 μg/mL) in addition to CD28 for 24 h. ConcavalinA (ConA) was used as a positive control. Proliferation was measured using MTT, which was added to the splenocytes at a final concentration of 0.5 mg/mL after 24 h of peptide stimulation. After 3 to 4 h, medium was removed, cells were treated with 100 μL dimethyl sulfoxide, and the absorbance was measured at 560 nm. The proliferation index was calculated by using the following formulas: proliferation index = (proliferation upon peptide stimulation)/(proliferation upon ConA stimulation); proliferation upon stimulation = (OD_stimulated_ − OD_unstimulated_)/(OD_unstimulated_).

### Inhibition of binding of labeled VLPs to Vero cells by immunized mouse sera.

The labeled VLPs were incubated with 1: 2 and 1:4 dilutions of serum for 1 h at 37°C. Vero cells (5 × 10^5^) were added to the mixture of SARS-CoV-2 VLPs and antibody in DMEM and 25 mM HEPES buffer (100 μL) and incubated for 2 h at room temperature. Unbound complexes were removed by washes. Cell-bound fluorescence was analyzed using a fluorescence-activated cell sorter (FACS) Verse flow cytometer (Becton Dickinson) and BDFACSuite software to calculate the cell population bound by VLPs, and percent binding was determined from the following equation: % binding of VLP to cells = [(% VLP bound to cells in experimental sample) − (% VLP bound in cells-only negative control)/(% VLP bound in no-sera positive control)] × 100.

### Immunofluorescence staining for FACS.

VLP was incubated with Vero cells at the described temperature for the indicated time. Unbound VLP was removed by washes. Cells were fixed with 4% paraformaldehyde for 20 min at room temperature. This was followed by PBS washes. Thereafter, cells were stained with nucleocapsid antibody (catalog number 40143-MM05, Sino Biological) for 1 h, followed by Alexa Fluor 633-conjugated secondary antibody. Cells were washed with PBS and analyzed using a FACS Verse flow cytometer (Becton Dickinson).

### Live virus neutralization.

Full-length SARS-CoV-2 (isolate Hong Kong/VM20001061/2020, NR-52282, BEI Resources, NIAID, NIH) was used for live virus neutralization (GISAID ID EPI_ISL_412028). The infectious virus was obtained from the IISc Viral BSL-3 repository. PBS and VLP immunized mouse sera were heat denatured at 56°C for 30 min and diluted in DMEM without serum. This mixture was incubated with the virus at 37°C for 1 h. This mix was added to Vero cell monolayers, seeded in 24-well plates, and incubated at 37°C for 1 h. Thereafter, the inoculum was removed and replaced with DMEM containing 0.8% agarose. The plates were incubated at 37°C for 48 h. After 48 h, 4% paraformaldehyde was used to fix the cells, which were stained with crystal violet to count the number of plaques. The ID_50_ of neutralization of VLP-immunized sera was calculated as the reciprocal of dilution at which the number of plaques was reduced to 50% of that observed in the PBS sera.

### Microscale thermophoresis.

ACE-2-Fc was a kind gift from Raghavan Vardarajan, IISc. The 2 μM AceII-Fc (in 1× PBS) was labeled using the RED-NHS Monolith protein labeling kit (NanoTemper Technologies) according to the manufacturer’s instructions. After labeling, the protein was eluted by gravity flow using a PD MiniTrapTM G-25 (GE Healthcare) Sephadex column in PBS. PBS was used as assay buffer for MST experiments. VLP concentration was calculated using the monomeric molecular weights of the structural proteins. WT-VLP, S mut-VLP, and S+N mut-VLP proteins were titrated in a 1:1 dilution series (highest concentrations ranged between 8 and 10 μM). Monolith NT.115 MST standard capillaries (NanoTemper Technologies) were loaded with the samples, and binding was measured with a Monolith NT.115 instrument using MO.Control software at room temperature (LED, excitation power setting 100%, MST power setting 80 to 100%). Data were analyzed using MO.Affinity analysis software (version 2.2.5, NanoTemper Technologies) at different standard MST-off times.

### Antibodies for entry inhibition.

The labeled VLPs were incubated with the mentioned dilutions of commercial antibodies for 1 h at 37°C. Antibod 1 (Ab1) (catalog number 40592-R001) and Ab2 (catalog number 40591-T62) were purchased from Sino Biological Inc. Vero cells (5 × 10^5^) were added to the mixture of SARS-CoV-2 VLPs and antibody in DMEM and 25 mM HEPES buffer (100 μL) and incubated for 2 h at room temperature. Unbound complexes were removed by washes. Cell-bound fluorescence was analyzed using a FACS Verse flow cytometer (Becton Dickinson) and BDFACSuite software to calculate the cell population bound by VLPs. Percent binding was determined from the following equation: % binding of VLPs to cells = [(% VLP bound to cells in experimental sample) − (% VLP bound to cells in cells-only negative control)]/[(% VLP bound to cells in no-antibody positive control) − (% VLP bound to cells in cells-only negative control)] × 100.
